# Supplementary figures and images for: ‘Intraoperative predictors for clinical outcomes after microinvasive glaucoma surgery”
Source: PLoS One. 2023 Nov 9;18(11):e0293212. doi: 10.1371/journal.pone.0293212 (PMC10635545; doi:10.1371/journal.pone.0293212)

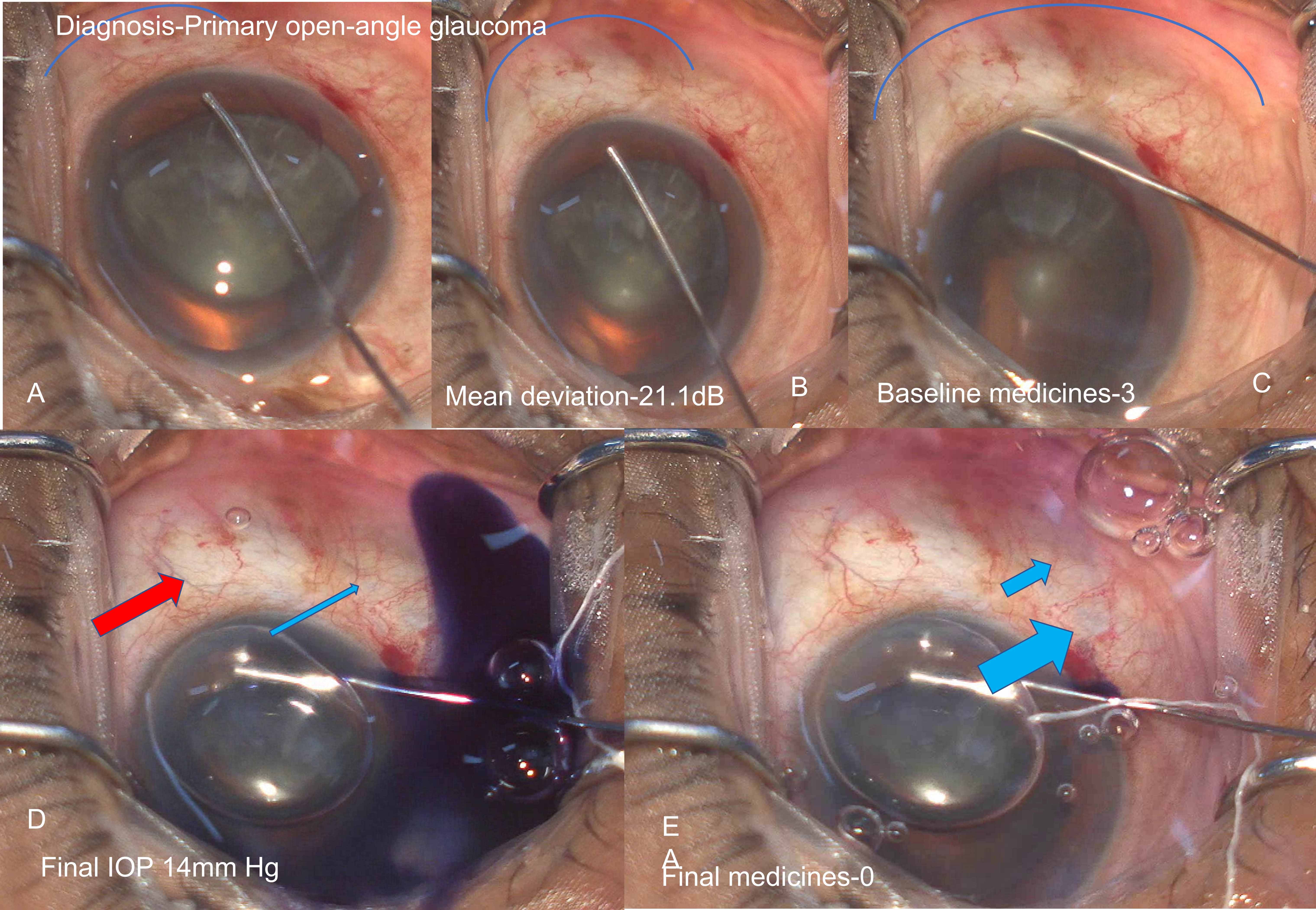

Supplement: S1 Fig — A-C shows the progressive blanching seen in 2 quadrants (arc) to 6 quadrants in C. The staining pattern in this eye was restricted to deep venular (red arrow) and blush haze (light blue arrow) staining in small regions in different quadrants. (JPG) [file pone.0293212.s001.jpg]
