# Supplementary material for: ‘Intraoperative predictors for clinical outcomes after microinvasive glaucoma surgery”
Source: PLoS One. 2023 Nov 9;18(11):e0293212. doi: 10.1371/journal.pone.0293212 (PMC10635545; doi:10.1371/journal.pone.0293212)
Supplement: S1 Table — See text for full description. (DOCX) [file pone.0293212.s002.docx]

Table S1: Comparison of the clinical profile of patients that underwent GATT or MIT

| Variables | GATT | MIT | P value |
| --- | --- | --- | --- |
| Baseline IOP (mm Hg) | 23±11.1 | 19±6.6 | 0.1 |
| IOP 1day (mm Hg) | 18±9.8 | 17±5.6 | 0.6 |
| IOP 2weeks (mm Hg) | 18±8.2 | 14±5.6 | 0.6 |
| IOP 1month (mm Hg) | 16±5.8 | 16±6.2 | 0.2 |
| IOP3month (mm Hg) | 19±7.1 | 16±6.7 | 0.9 |
| IOP6 months (mm Hg) | 14±7.6 | 15±5.8 | 0.6 |
| IOP1 year (mm Hg) | 15±6.5 | 13±3.4 | 0.7 |
| Final IOP (mm Hg) | 16±5.2 | 14±3.5 | 0.5 |
| Number of medicines | 0.4±0.8 | 0.2±0.6 | 0.1 |

IOP-intraocular pressure;
