# Supplementary material for: ‘Intraoperative predictors for clinical outcomes after microinvasive glaucoma surgery”
Source: PLoS One. 2023 Nov 9;18(11):e0293212. doi: 10.1371/journal.pone.0293212 (PMC10635545; doi:10.1371/journal.pone.0293212)
Supplement: S2 Table — (See text for full description). (DOCX) [file pone.0293212.s003.docx]

Table S2: Comparisons of blanching and blue staining for intraoperative outflow assessment after GATT or MIT (See text for full description).

| Blanching  N=167 | GATT  N=49 | MIT  N=118 |
| --- | --- | --- |
| Age  Baseline IOP  Baseline MD | 53±20.7  23±11.1  -17±10.1 | 59±12.8  19±6.6  -16±8.8 |
| Quadrants seen | 0 1  1 2  2 26  3 13  4 2  5 5  6 0  8 0 | 0 12  1 6  2 43  3 23  4 15  5 7  6 11  8 1 |
| Trypan blue staining  N=99 | GATT  N=7 | MIT  N=92 |
| Quadrants seen | 0 5  1 1  2 1  3 0  4 0  5 0 | 0 21  1 12  2 33  3 10  4 6  5 10 |

MIT-Micro incisional trabeculectomy; GATT- gonioscopy-assisted transluminal trabeculotomy
