# Supplementary material for: ‘Intraoperative predictors for clinical outcomes after microinvasive glaucoma surgery”
Source: PLoS One. 2023 Nov 9;18(11):e0293212. doi: 10.1371/journal.pone.0293212 (PMC10635545; doi:10.1371/journal.pone.0293212)
Supplement: S3 Table — See text for full description. (DOCX) [file pone.0293212.s004.docx]

Table S3: Multivariate regression of clinical variables predicting the need for medicines after GATT or MIT-see text for full description.

|  | β (regression coefficient) | P value |
| --- | --- | --- |
| Age | -0.11 | 0.2 |
| Baseline IOP | -0.03 | 0.1 |
| Baseline MD | 0.02 | 0.2 |
| Quadrants of incision | -0.1 | 0.4 |
| Quadrants of blanching <2 | 0.2 | 0.03 |
| Quadrants of blue staining<2 | 0.09 | 0.04 |

MD-Mean deviation, IOP-intraocular pressure
