# Supplementary material for: ‘Intraoperative predictors for clinical outcomes after microinvasive glaucoma surgery”
Source: PLoS One. 2023 Nov 9;18(11):e0293212. doi: 10.1371/journal.pone.0293212 (PMC10635545; doi:10.1371/journal.pone.0293212)
Supplement: Attachment — Submitted filename: response to reviewer cover letter1.docx [file pone.0293212.s006.docx]

­­To,

The Editor,

Dear Sir/Madam,

We are pleased to submit our revised manuscript titled "Intraoperative Predictors for Clinical Outcomes after Microinvasive Glaucoma Surgery." Additionally, we have had the manuscript professionally edited. We have taken care to address each of the reviewers' comments with detailed point-to-point clarifications. We remain open to any further suggestions or feedback that could contribute to enhancing the quality of the manuscript. Thank you for your consideration.

All the authors have contributed equally towards the preparation of the manuscript and have no financial or proprietary interest in the products used in the study. We also declare that this article has not been published previously or is under review with any other journal.

a) All acknowledgments and financial disclosures/funding information is included in the manuscript. We would like to clarify that this acknowledgment is for the research foundation supporting all the institute's healthcare-related studies. This does not entail funding from the foundation. This study did not receive any funding from any agency or organization.

b) All data have been given in the manuscript with additional patient-identifying information that may be shared after consent upon request. We have also made available a minimal dataset along with all relevant data that has already been shared in the supplemental data.

Thanking you

1. Please ensure that your manuscript meets PLOS ONE's style requirements, including those for file naming. The PLOS ONE style templates can be found at

https://journals.plos.org/plosone/s/file?id=wjVg/PLOSOne_formatting_sample_main_body.pdf and

<https://journals.plos.org/plosone/s/file?id=ba62/PLOSOne_formatting_sample_title_authors_affiliations.pdf>

Answer; we do confirm the same

2. Please include your full ethics statement in the ‘Methods’ section of your manuscript file. In your statement, please include the full name of the IRB or ethics committee who approved or waived your study, as well as whether or not you obtained informed written or verbal consent. If consent was waived for your study, please include this information in your statement as well.

Answer: This has already been provided, as per instructions.

3. We notice that your supplementary figure is uploaded with the file type 'Figure'. Please amend the file type to 'Supporting Information'. Please ensure that each Supporting Information file has a legend listed in the manuscript after the references list.

Answer: we have changed this now to supporting information, as suggested.
Additional Editor Comments:

It is a well written manuscript describing the predictors for intraoperative success after MIGS.
The criteria for qualified success/ failure is not clear. The authors have mentioned maximum tolerable glaucoma medications. Did they count only topical medications or also counted oral medications in defining qualified success?
In the results section, last paragraph, the authors mentioned that intraoperative stain in > 2 quadrants was associated with higher number of medications. It should be vice a versa.
The authors have commented on final follow up while describing results. They should mention if they are mentioning results at 1 year follow up, or should define what was the mean and median follow up if they want to write the final follow up.

Answer: We deeply appreciate the insightful points of the editor that we have now addressed in the revised manuscript. Additionally, we have clarified the criteria for success to eliminate any potential confusion for readers. Moreover, we have incorporated the suggested information regarding the final follow-up duration. Thank you for your valuable guidance.

Reviewers' comments:

5. Review Comments to the Author

Reviewer #1: Thank you for submitting your article. Overall, I found the paper to be well-written and informative. However, I have a few suggestions that I believe would improve the quality of the article. Please address these minor corrections before resubmitting your work. Overall, I believe these minor corrections will enhance the clarity and impact of your article.

Answer: We express our gratitude to the reviewer for their encouragement and sincerely appreciate their valuable suggestions. We welcome any further recommendations to enhance the manuscript and are truly thankful for the significant improvements made by incorporating these insightful suggestions.

Reviewer #2: The author launch an interesting study about MIT and GATT, which want to find the predictor factor of these MIGS proceduere. The details of the study, statistical methods, still need to be clarified before the article is published:
1) According to the author's narratives in limitation paragraphs, GATT is 360 degree trabcular meshwork cutting while MIT is a quadrant incision method. The blanching or staining difference between these two group should be analized in the manuscript. And the result of surgical success ( complete or qualified ) should also comparing between the two groups.

Answer: We certainly concur that a comparison of the blanching or staining method between the two approaches would be beneficial. Furthermore, it's important to highlight that we discovered no discernible difference in blanching patterns between GATT and MIT (the same reflected in the multivariate analysis with the type of surgery not defining the blanching or blue staining pattern). However, we wish to clarify that our intention was not to conduct a direct comparative study between GATT and MIT. Instead, our focus was on developing a simplified method for assessing the outflow pathway. A comparative analysis between these two surgeries, considering the variation in the number of quadrants operated, could introduce confounding factors. For instance, a greater extent of deroofing is logically expected to lead to a more substantial reduction in outflow resistance though this was not seen in this study. This consideration influenced our decision not to pursue a comparative study in this research. This information has already been detailed in the results section (outflow assessment subheading), indicating that a comparative analysis of this nature would yield similar outcomes. Yes, we are comparing the prospective clinical outcomes between GATT and MIT-we would be delighted to incorporate this suggestion to see the differences in blanching/blue staining patterns with other anterior segment imaging parameters in that study.

2) The author use multivariiate regression module to analysis the predict facotr of surgical success. But the details table of this statistical analysis is not presented in the manuscript.

Answer: We wish to provide clarity regarding the inclusion of multivariate regression details and considered variables. These specifics have been outlined in the manuscript's Results section, specifically in the final paragraph. We are more than willing to share any additional information pertaining to this matter. Given that a significant association was not observed for most variables, we made the decision not to present this information in a table or figure and rather included the details in the text.

3) Whether the set position of the protractor is fixed, or based on the TM incision position (especially in MIT surgery), or the position where the irrigation needle is placed ? If it is only a partial incision (MIT), then the quadrants of blanching or staining will actually be affected by the incision site.

Answer: We want to provide further clarification regarding the protractor tool mentioned in our study. It's important to note that the protractor tool we utilized is a virtual online tool that is superimposed onto the image or video. It doesn't involve physically placing the tool on the eye during surgery. This tool is positioned over the eye in a manner that spans from limbus to limbus. Consequently, its placement is not influenced by factors such as the incision site or any similar considerations.
