# Supplementary material for: ‘Intraoperative predictors for clinical outcomes after microinvasive glaucoma surgery”
Source: PLoS One. 2023 Nov 9;18(11):e0293212. doi: 10.1371/journal.pone.0293212 (PMC10635545; doi:10.1371/journal.pone.0293212)
Supplement: Attachment — Submitted filename: Review.docx [file pone.0293212.s007.docx]

The article is well-organized and presents a valuable contribution to the field of microinvasive glaucoma surgery. The methodology is robust, but potential limitations, such as the retrospective nature of the study and the sample size, are appropriately acknowledged. The discussion effectively connects the findings to broader clinical implications.

Note:

A few grammatical errors in the article have been identified.

Original Text: "All eyes had a significant reduction in the number of medications after surgery."

Suggested Correction: "All eyes experienced a significant reduction in the number of medications after surgery."

Original Text: "The means of evaluating the outflow system include aqueous angiography, fundus fluorescein angiography, aqueous angiography, 3-D visualization, and optical coherence tomography angiography."

Suggested Correction: "The means of evaluating the outflow system include aqueous angiography, fundus fluorescein angiography, 3-D visualization, and optical coherence tomography angiography."

Original Text: "Trypan blue is a cheap and effective alternative means of assessing the outflow under direct visualization after the surgery under the operating microscope."

Suggested Correction: "Trypan blue offers a cost-efficient and viable option for assessing outflow under direct visualization following surgery with the aid of the operating microscope."
