# Supplementary material for: ‘Intraoperative predictors for clinical outcomes after microinvasive glaucoma surgery”
Source: PLoS One. 2023 Nov 9;18(11):e0293212. doi: 10.1371/journal.pone.0293212 (PMC10635545; doi:10.1371/journal.pone.0293212)
Supplement: Attachment — Submitted filename: PLos reply to comments R2.docx [file pone.0293212.s008.docx]

­­To,

The Editor,

Dear Sir/Madam,

We are pleased to submit our revised manuscript titled "Intraoperative Predictors for Clinical Outcomes after Microinvasive Glaucoma Surgery." Additionally, we have had the manuscript professionally edited. We have taken care to address each of the reviewers' comments with detailed point-to-point clarifications. We remain open to any further suggestions or feedback that could contribute to enhancing the quality of the manuscript. Thank you for your consideration.

All the authors have contributed equally towards the preparation of the manuscript and have no financial or proprietary interest in the products used in the study. We also declare that this article has not been published previously or is under review with any other journal.

a) All acknowledgments and financial disclosures/funding information is included in the manuscript. We would like to clarify that this acknowledgment is for the research foundation supporting all the institute's healthcare-related studies. This does not entail funding from the foundation. This study did not receive any funding from any agency or organization.

b) All data have been given in the manuscript with additional patient-identifying information that may be shared after consent upon request. We have also made available a minimal dataset along with all relevant data that has already been shared in the supplemental data.

Thanking you

Associate editor:

The authors have done a great job in addressing the comments. However they are advised to describe the results of GATT and MIT separately in Table 1 and 2, even if they want to avoid comparative analysis. Because the eyes with trypan blue stain and blanching would be different in eyes with MIT and GATT, due to difference in quadrants with trabecular meshwork excusiom. Therefore it would be nice to present the results of two groups differently. Its the author's choice if they don't want to compute statstical significant results between two groups or not, since that is not the primary intention of the study.

Answer: We would like to clarify that as stated in the manuscript, trypam blue was done only after June so included only 99 eyes. We have therefore presented the blanching/staining between the GATT and MIT eyes in a supplementary table for logistic reasons of including that information in table 1 or 2 and to avoid confusion for readers when talking about outflow assessment in general.

Review Comments to the Author
Reviewer #1:

The article is well-organized and presents a valuable contribution to the field of microinvasive glaucoma surgery. The methodology is robust, but potential limitations, such as the retrospective nature of the study and the sample size, are appropriately acknowledged. The discussion effectively connects the findings to broader clinical implications.

Answer: We thank the reviewer for the approval and would welcome any further suggestions.
:
A few grammatical errors in the article have been identified.
Original Text: "All eyes had a significant reduction in the number of medications after surgery."
Suggested Correction: "All eyes experienced a significant reduction in the number of medications after surgery."
Original Text: "The means of evaluating the outflow system include aqueous angiography, fundus fluorescein angiography, aqueous angiography, 3-D visualization, and optical coherence tomography angiography."
Suggested Correction: "The means of evaluating the outflow system include aqueous angiography, fundus fluorescein angiography, 3-D visualization, and optical coherence tomography angiography."
Original Text: "Trypan blue is a cheap and effective alternative means of assessing the outflow under direct visualization after the surgery under the operating microscope."
Suggested Correction: "Trypan blue offers a cost-efficient and viable option for assessing outflow under direct visualization following surgery with the aid of the operating microscope."

Answer: All the grammatical corrections have been made, as suggested by the reviewer.

Reviewer #2:

1）Since the author has listed two different surgical methods, the results should still be presented according to different surgical types, rather than mixed together. Regardless of whether there is a statistical difference between the two groups, the relevant data in Table 1 and 2 needs to be clearly presented to the reader.

Answer: We have included a separate table S2 on results for GATT and MIT with regards to blanching since blue staining was done only after June 2022 (n=99 eyes).

2）The results of the multi variate regression analysis also need to be presented, even in the appendix section.

Answer: As suggested, we have now included a supplementary table S3 on the multivariate analysis.

3）Why choose p<0.02 as the judgment criterion in the statistical method section. In general, this standard is either p<0.01 or p<0.05.

Answer: While doing a multivariate analysis, it is preferable to include only those variables that have a significant association on a univariate analysis that makes the statistical comparison robust. In this procedure, we include only those that show a significant association on univariate rather than p<0.02 so that spurious association on a multivariate model is avoided which is not uncommon if we include a lot of univariate variables with weak associations (It may be common that p<0.05 may give significance ion multivariate model while variables on univariate may not be statistically significant). This is the reason we also chose p<0.02 as that mark for inclusion into the multivariate model.

4）Regarding this question of "3) Why the set position of the predictor is fixed, or based on the TM decision position (specifically in MIT summer)...", I would like to know whether the author judges it based on fixed quadrants or hours site? If the range of blank is 5-7 hours, it is 3 hours (equivalent to a quadrant) calculated by hour position; If calculated based on fixed quadrants, it is 2 quadrants.

Answer: We agree and clarify that all the comparisons were made for quadrants and not clock hours as stated in the manuscript.
